# Controlled gonad development: effects of environmental factors on sexual maturity in red swamp crayfish, *Procambarus clarkii*

**DOI:** 10.3389/fphys.2026.1956361

**Published:** 2026-10-15

**Authors:** Jian-gang Jiao, Biao Yun, Xue-qiao Qian

**Affiliations:** Guangdong Haid Group Co., Ltd., Guangzhou, China

**Keywords:** aquaculture, crayfish, light intensity, temperature, vitellogenin

## Abstract

Because development of crustacean gonads can impede growth potential, a balance between growth and gonad development is necessary to promote the healthy and sustainable development of aquaculture. We report growth and gonad development in red swamp crayfish *Procambarus clarkii* at six temperatures between 16 °C and 36 °C, and conduct experiments to investigate the impact of light intensity on growth, gonad development, and survival at 36 °C. Growth in crayfish first increased, then decreased with increased temperature. Above 28 °C, 10%–30% of gonads were mature, but the survival rate of crayfish above 28 °C was significantly lower than that below 28 °C. Hemolymph vitellogenin (VTG) content increased significantly with increased temperature; expressions of vtg gene and molt-inhibiting hormone (MIH) in hepatopancreatic and ovarian tissues gradually increased; and expression of vitellogenesis-inhibiting hormone decreased significantly. At 36 °C, crayfish treated with 400–500 lx light (50% relative light intensity, RLI) showed significantly higher weight gain and survival rates than controls (800–1,000 lx, 100% RLI), and gonad development was slightly lower; hemolymph VTG contents of treatment crayfish were lower than that of controls; vtg expression decreased significantly in the ovary but not in the hepatopancreas; expression of vitellogenesis-inhibiting hormone in the hepatopancreas gradually increased; and expression of molt-inhibiting hormone did not differ significantly. Therefore, growth promotion and delayed gonad development can be achieved by decreasing light intensity during crayfish culture at high temperature, reducing VTG accumulation rates in ovaries.

## 1 Introduction

Red swamp crayfish (*Procambarus clarkii*) are commercially important in freshwater aquaculture in China. The Food and Agriculture Organization of the United Nations (FAO) (FAO, 2022) reported that in 2020, 96.8% of the 2.469 × 106 t of crayfish globally farmed was farmed in China (CSF, 2022). As China’s second-largest freshwater-farmed crustacean by production, crayfish lack the same sophisticated artificial breeding and factory farming models as white shrimp (*Litopenaeus vannamei*), and their farmed growth is severely constrained. During the breeding period, crayfish burrow into the sediment, and with sexual maturation, their growth declines. Therefore, in pond culture, crayfish must be harvested before sexual maturity, mainly between April and June, because from July to September, temperatures are high, and many crayfish reach sexual maturity, greatly affecting the breeding effect (Ando, 1998; Shui et al., 2020). In the context of global warming, the increasing average temperature in the Yangtze River basin year by year will also exacerbate this issue. Because the control of neuroendocrine hormone levels in giant freshwater prawn (*Macrobrachium rosenbergii*), also a crustacean, can inhibit gonad development and promote growth (Megawati et al., 2020), a similar solution may delay sexual maturation in crayfish during summer.

Crustaceans have a unique set of mechanisms that regulate gonad development. Crustacean hyperglycemic hormone (CHH) secreted by the X-organ-sinus gland (XO–SG) complex in the eyestalk regulates gonad development, molt growth, and other physiological processes (Qian et al., 2009). While eyestalk ablation can promote molting and gonad development (Manfrin et al., 2015), a non-invasive technical solution is preferred to regulate crayfish molt and gonad development (Chaves, 2001). One regulatory pathway includes secretion of farnesoic acid (FA), one of the more specific hemiterpenes in crustaceans, from the Y-organ in the maxillary apparatus under the regulation of, for example, CHH secreted by the XO–SG. The secreted FA can then be further oxidized to active forms that regulate ovarian development and molting, or directly through, for example, vitellogenesis-inhibiting hormone (VIH), which regulates yolk proteinogen synthesis in the hepatopancreas or ovaries and thus their development (Chung et al., 2010). Studies on American lobster (*Homarus americanus*) and white shrimp (*L. vannamei*) have indicated that elevated expression of the gonad-inhibiting hormone (GIH) gene belonging to the CHH family leads to a considerable decrease of ovarian vitellogenin (VTG) levels, hampering reproduction (Soyez et al., 2010; Kang et al., 2014). Consequently, effective control of gonad development in crayfish could be achieved through regulation of the CHH signaling pathway.

Numerous studies have reported environmental factors to significantly affect crustacean molt and gonad development (Abdu et al., 2001). Light, temperature, dissolved oxygen, and salinity can all regulate the XO–SG complex (Nagaraju, 2010). An appropriate temperature range is conducive to crustacean growth and gonad development. Qiao et al. reported GIH expression in Japanese marsh shrimp (*Macrobrachium nipponense*) to increase and then decrease with increased temperature (Qiao et al., 2015). Gonad development in crayfish also increased with increased temperature, with 17% of gonads developing to previtellogenic stage at 16 °C, but 53% of gonads developing to vitellogenic and mature stages at 26 °C (Carmona-Osaldea et al., 2004). Eyestalk tissue is light-sensitive, and photoperiod regulates secretions from neuronal cells (Vargas et al., 2010). Japanese lobster ovaries have been shown to develop faster and have the optimum egg-holding rate at 25 °C with a 14L:10D photoperiod (Matsuda et al., 2002). More advanced gonad development was also reported for fairy lobster under summer photoperiod conditions as opposed to winter photoperiod conditions (Sachlikidis et al., 2005). However, the effects of light intensity on crustaceans are less well studied.

We report body weight growth and sexual maturity development in red swamp crayfish between 16 °C and 36 °C (naturally experienced during spring to summer in crayfish pond water in Hubei Province, China), and investigate the effects of light intensity on growth, gonad development, and survival at 36 °C. To investigate relationships between habitat conditions and endocrine regulation, and to identify a feasible integrated solution (including nutritional and physical approaches) to delay the onset of gonad development and maintain growth at high temperatures, we also examine gene expression of the CHH family of hormones secreted by the XO–SG complex when stimulated by different environmental factors.

## 2 Materials and methods

### 2.1 Test animals

Juvenile female red swamp crayfish (6.2 g ± 0.7 g) were obtained from an aquafarm in Huangshi, China. All crayfish were temporarily reared in indoor concrete ponds (5 m × 5 m, with several artificial aquatic mats [polyethylene, 0.5 m × 0.5 m per mat] provided as shelter) for 5 d in April 2024 (see **Figure 1** for a schematic diagram). In order to avoid crayfish burns from the heater, we conducted the experiment by placing 30 crayfish from each pond equally into two 1 m × 1 m net cages. Culture water was a mixture of pond purification and heated water from outside ponds and groundwater wells, with fixed parameters: dissolved oxygen (5–7 mg/L), temperature (16 °C ± 2 °C), pH (7.4–8.1), and total ammonia-nitrogen (<0.07 mg/L). After 5 days of acclimation, crayfish were transferred separately into the experimental system (each tank as a replicate containing 30 crayfish), with three replicate tanks per experimental treatment. Crayfish were fed a commercial feed (crude protein percentage = 30) twice daily (7:00 and 17:00) at 2%–3% of their body weight.

**Figure 1:**
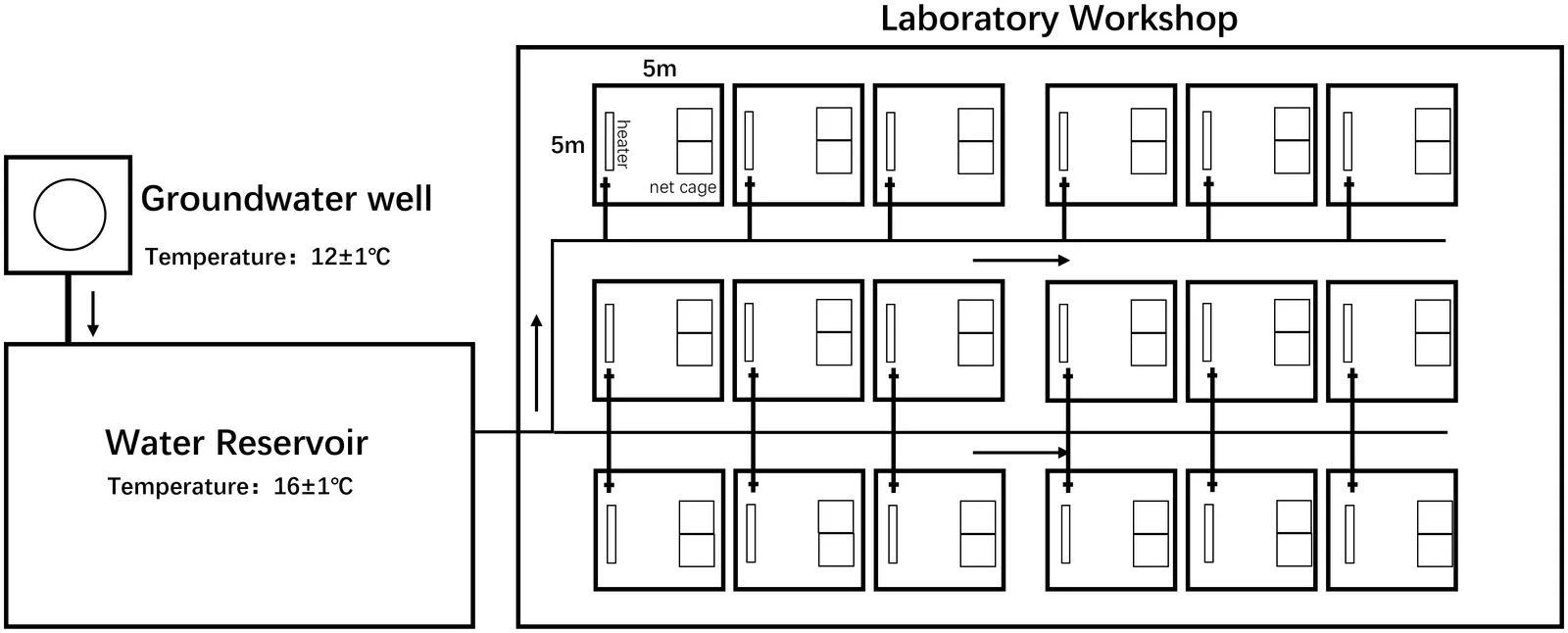
Sketch of aquaculture pilot plant and equipment. The direction of the arrow in the diagram is the direction of water flow.

Based on the ovarian color change and appearance characteristics, the ovarian development of crayfish was classified into five stages: I, non-development: white transparent, no granular eggs; II, early developmental: tiny white translucent oocytes; III, previtellogenic: uniform grayish-yellow or yellow eggs (10–300 μm in diameter); IV, vitellogenic: yellowish brown or dark brown eggs (450 μm–1.6 mm in diameter); V, mature: dark brown eggs (>1.5 mm in diameter) (Sarojini et al., 1995). Staging was performed by a single trained observer blinded to treatment identity, following the color- and morphology-based classification adopted in previous molecular studies of this species (Manfrin et al., 2021; Zhong et al., 2021).

All experimental procedures were performed in strict accordance with the Management Rule of Laboratory Animals (Chinese Order No. 676 of the State Council, revised 1 March 2017).

### 2.2 Experimental protocol

(I) To investigate growth (final total body weight of crayfish, FW) and gonad development (staging of ovary development in the crayfish) at different temperatures, six temperature treatments (16, 20, 24, 28, 32, and 36 °C) with commercial feed were established for 60 d. For the temperature acclimation process following the transfer of juvenile crayfish into the experimental system, this experiment employed a gradient ramping method to gradually adjust the temperature of each group to its designated target level. As shown in the schematic diagram (**Figure 1**), lower-temperature well water was first transported to the experimental pond through a water pipe, and then the water was gradually heated to the corresponding experimental temperature by a temperature-controlled heater in the pond (on average, the water temperature increased by 2 °C every 24 h). This ramping phase was included within the 60-day experimental period; once the target temperature was reached, each group was maintained at that temperature until the end of the experiment, so that all groups experienced a maximum of 10 days at their final target temperature. The water was changed by 1/10 per day, and the water was added more slowly to ensure that the water temperature did not fluctuate dramatically. After weighing at the end of the experiment, crayfish hemolymph was extracted using a disposable sterile syringe (1 mL) and transferred into 1 mL polyethylene tubes and left at room temperature for 10 min before refrigeration at 4 °C. After confirming the stage of ovarian development (Sarojini et al., 1995), the hemolymph, hepatopancreas, ovary, and eyestalk were separated and placed at −80 °C for RNA extraction.

To test VTG content, thawed hemolymph was centrifuged in a refrigerated centrifuge (4 °C, 20,000×*g*, 15 min) to obtain the supernatant, which was then assayed using a Prawn vitellogenin (VTG) ELISA Kit (Shanghai Yanjin Biotechnology Co., Ltd., China).

(II) To investigate the effect of light intensity and nutritional intervention on growth and gonad development at 36 °C, we established one control and two reduced light-intensity treatments for crayfish exposed to artificial light (fluorescent lamps) by adjusting the number of layers of shading polyethylene netting (0, 2, or 4 layers) (control, 800–1,000 lx as 100% relative light intensity (RLI), and at 50% [400–500 lx] and 20% [150–200 lx] of this value), and a 12:12 light/dark cycle. This light intensity gradient was set to simulate light under shade in ponds during high-temperature periods. Gonads were staged, and tissue was isolated, as for experiment (I). Light intensity at the water surface was measured daily at four fixed positions per tank with a digital illuminometer (UT383, UNI-T, China); when readings deviated by >10% from the target range, the number of shading-net layers or the lamp height was adjusted. The light/dark cycle was controlled by automatic timers, and water temperature, dissolved oxygen, pH, and total ammonia-nitrogen were monitored daily and did not differ significantly among the light treatments (P >0.05).

### 2.3 RNA isolation and real-time quantitative PCR

Samples of hepatopancreas, ovary, and eyestalk from each treatment underwent RNA extraction. Total RNA extraction utilized Trizol reagent (RN0101, Aidlab Biotechnologies Co., Ltd., Beijing, China). The quantity and quality of the RNA were assessed using a NanoDrop 2000 spectrophotometer (Thermo Fisher Scientific, Wilmington, USA). Total RNA (1,000 ng) was reverse transcribed utilizing a PrimeScript RT Master Mix (RR047A, Takara Bio Inc., Shiga, Japan) at a temperature of 42 °C for a period of 2 min with the aim of removing genomic DNA, and at 37 °C for a duration of 15 min and 85 °C for a period of 5 s with the objective of completing the reverse transcription process. Following this, the cDNA concentration was adjusted in accordance with the measured RNA concentration prior to the implementation of reverse transcription-PCR (RT-PCR). Reverse transcription-polymerase chain reaction (RT-PCR) amplification was performed utilizing 2× ultra SYBR mixtures (CW0957, Kangwei, China). The glyceraldehyde-3-phosphate dehydrogenase (GAPDH) gene was employed as the internal control reference gene; the GAPDH primer pair was adopted from Jiang et al. (2015), and GAPDH has been used to normalize VTG-family transcript levels in this species (Manfrin et al., 2021). The primers employed for the amplification of metabolic genes associated with gonad development are listed in **Table 1**.

**Table 1:** Primer sequences of metabolic genes associated with gonad development genes in hepatopancreatic and gonadal tissues.

Target gene	Forward primer sequence	Reverse primer sequence
*gapdh*	GCCCAGAACATCATCCCATCT	CGTCATCCTCAGTGTAACCCAAG
Vitellogenin *(vtg)*	CCGGCTGAGCTTCACTAACC	AGTCGATGCGGTTGCCTTAT
vitellogenesis-inhibiting hormone *(vih)*	GAATGTCCTGGAGTGATGG	CAGCGTTAAGAATACCAATCC
molt-inhibiting hormone *(mih)*	AGAGTGACAAGCCTTCCA	ACAGTGCGACAGTTGATG

The preparation of the polymerase chain reaction (PCR) samples was conducted in a volume of 10 µL. The composition of this volume was as follows: 5 µL of 2× ultra SYBR mixture, 0.25 µL of 10 mM forward and reverse primers, 2 µL of cDNA template, and 2.5 µL of H2O. The preparation was conducted under the following conditions: initial denaturation at 95 °C for 30 s, followed by 40 cycles of 94 °C for 15 s and 72 °C for 10 s in a CFX96 Real-time PCR system (Bio-Rad, Richmond, CA, USA). Each sample was amplified in triplicate (technical replicates) and the mean Ct value was used for analysis; no-template and no-reverse-transcription controls were included on every plate, and a single specific amplicon was confirmed by melt-curve analysis and agarose gel electrophoresis. Amplification efficiencies of all primer pairs were 96%–104% (R² >0.99), estimated from standard curves generated with serially diluted cDNA. Relative expression was calculated with the 2−ΔΔCt method, with the 16 °C group (temperature experiment) or the 100% RLI group (light experiment) as the calibrator. Total RNA was extracted from the whole eyestalk; because the X-organ-sinus gland (XO–SG) complex is the principal site of vih and mih transcription within the eyestalk, whole-eyestalk RNA is expected to reflect XO–SG expression, although the XO–SG was not microdissected.

### 2.4 Statistical analysis

The tank was the experimental unit; each treatment comprised three replicate tanks (n = 3), each holding two 1 m × 1 m net cages (15 crayfish per cage) to reduce cannibalism. At the end of the experiment, crayfish were sampled at random from both net cages of each tank, and the two cage values were averaged to give a single value per tank for VTG content, gene expression, and gonad stage; final body weight was recorded at the tank level. Results are expressed as means ± SEM (n = 3 tanks per treatment). Survival rate and gonad developmental-stage proportions were arcsine square-root transformed before analysis, and homogeneity of variance was verified with Levene’s test (Wang et al., 2022; Ly et al., 2024). One-way analysis of variance (ANOVA) followed by Duncan’s multiple range test was used to identify significant differences (P <0.05) in growth, VTG content, and related gene expression between temperatures and light intensities. All analyses were conducted using the IBM SPSS Statistics 19.0 package.

## 3 Results

### 3.1 Crayfish growth and gonad development at different temperatures

Temperature significantly affected crayfish growth (**Figure 2A**). With a gradual increase in temperature, crayfish weight increased significantly (P <0.05), but declined above 28 °C, and decreased significantly at 36 °C (P <0.05). Temperature also affected survival rates and gonad development (**Figure 2B**). Crayfish survival rate decreased significantly when the water temperature was higher than 28 °C (from 96.7% ± 3.3% to 72.2% ± 7.7%) (**Figure 2B**). The effect of temperature was mainly manifested in proportions of different gonad developmental stages; at <24 °C, the proportion of non-development (Stage I) gonads exceeded 85%, and there were no signs of gonad development; a small proportion of gonads were previtellogenic (Stage III) at 28 °C. Above 28 °C, 10%–30% of gonads were mature, and at 36 °C, few undeveloped crayfish remained.

**Figure 2:**
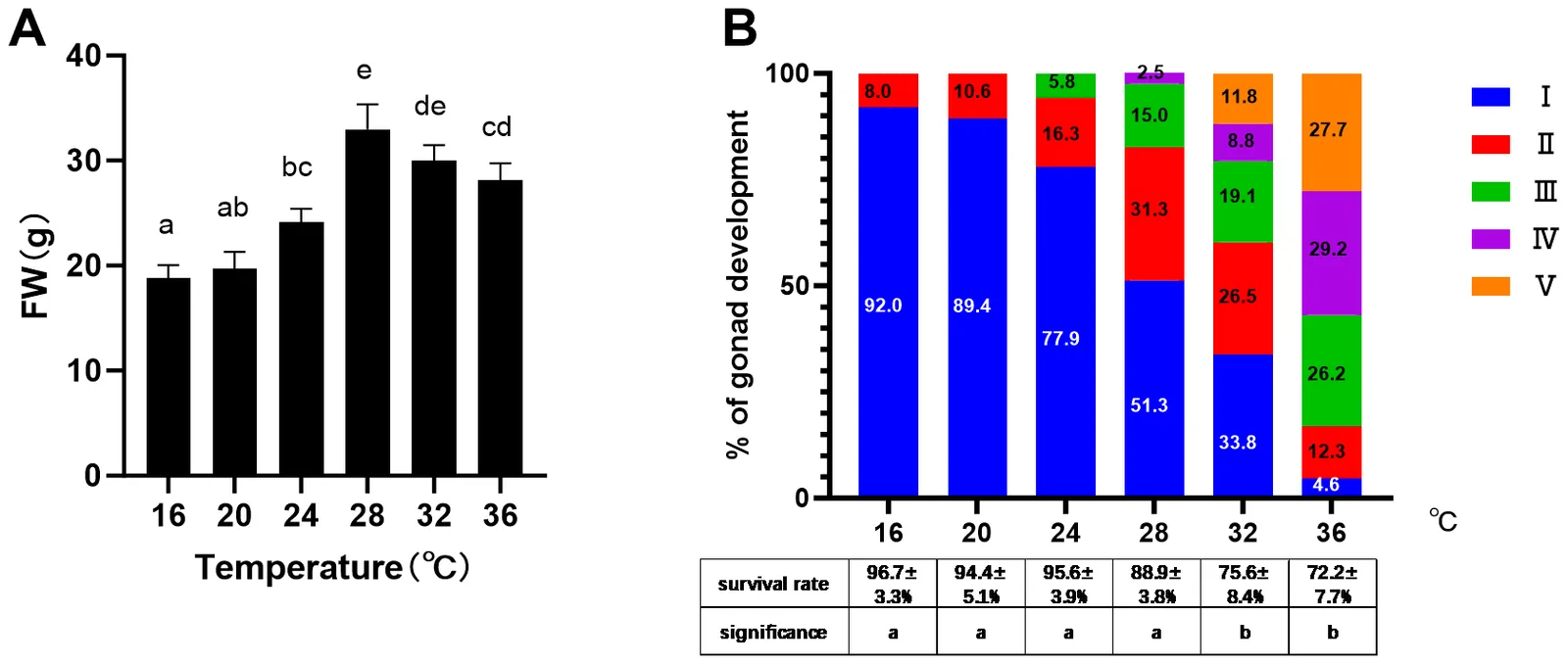
Effect of temperature on growth **(A)** and gonad development and survival rate **(B)** in crayfish, *Procambarus clarkii*. Values in **(A)** are from three replicate tanks per treatment (n = 3) (mean ± SEM); in **(B)** ovary stage (I, non-development; II, early developmental; III, previtellogenic; IV, vitellogenic; V, mature). Different letters indicate significant differences, P <0.05.

### 3.2 Crayfish VTG metabolism at different temperatures

From 16°C to 36 °C, VTG concentrations in hemolymph gradually increased, and were significantly higher from 32°C to 36 °C compared with concentrations at lower temperatures (P <0.05) (**Figure 3A**). Expression of vtg mRNA was significantly higher in the hepatopancreas than in the ovary at all temperatures (**Figures 3B, C**), and increased significantly with increasing temperature in both hepatopancreatic and gonadal tissues (P <0.05), with gene expression at 32–36 °C being 10–40× higher than that below 24 °C (**Figures 3B, C**). VIH and molt-inhibiting hormone (MIH), secreted in the XO–SG complex, regulate VTG metabolism in the hepatopancreas and ovary. At temperatures >24 °C, VIH mRNA expression decreased significantly (P <0.05), while MIH mRNA expression increased significantly (P <0.05); there were no significant difference in the range 16–24 °C in either VIH and MIH expressions (**Figures 3D, E**).

**Figure 3:**
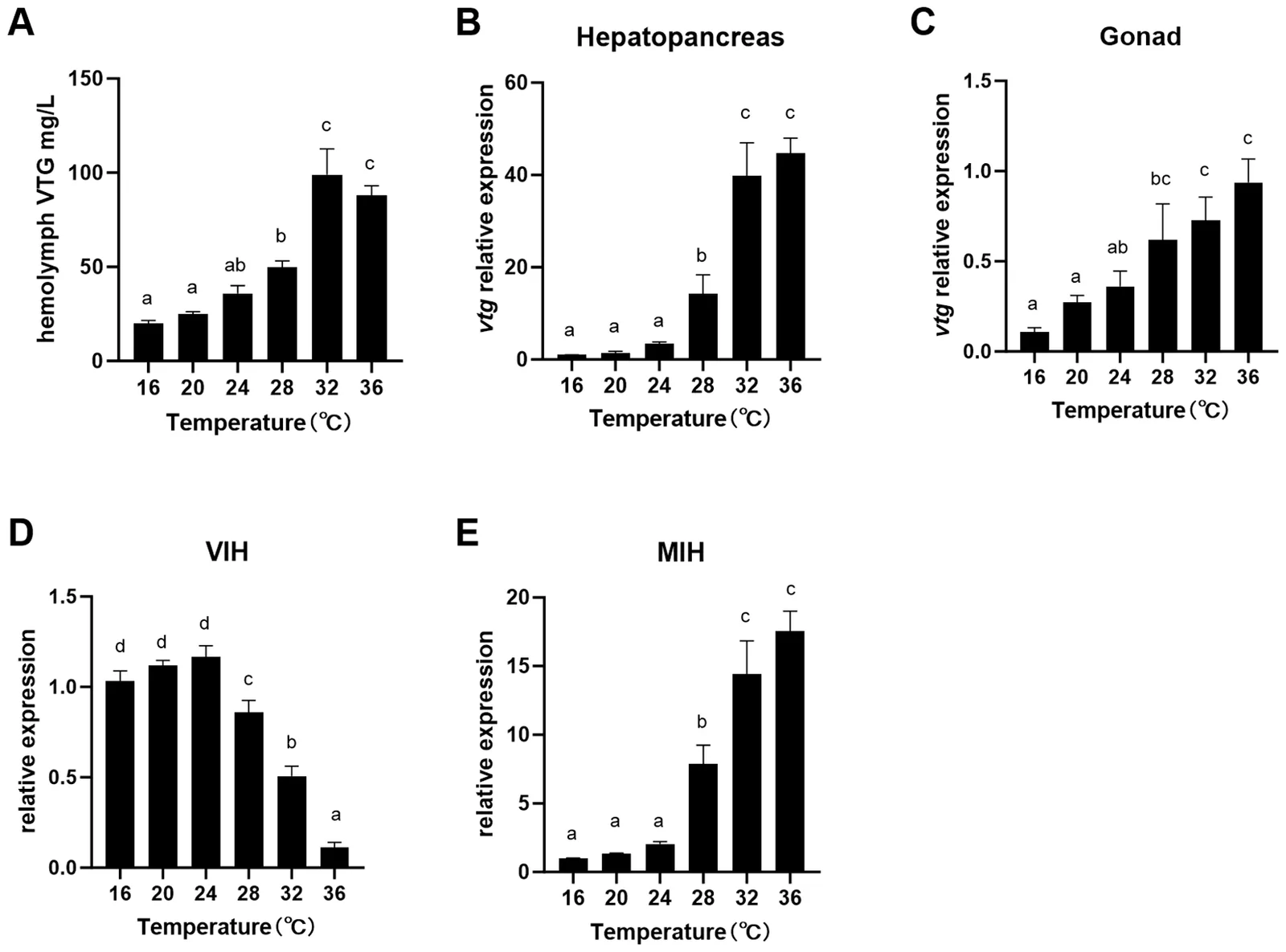
Effect of temperature on VTG metabolism in crayfish, *Procambarus clarkii*: **(A)** effect of temperature on hemolymph VTG content; **(B,C)** vitellogenin (*vtg*) relative expression in hepatopancreatic **(B)** and gonadal **(C)** tissue, which in the hepatopancreas at 16 °C was set to 1; **(D, E)** relative expressions of vitellogenesis-inhibiting hormone (*vih*) and molt-inhibiting hormone (*mih*) in the XO–SG complex compared to the amounts expressed at 16 °C. All values were from three replicate tanks per treatment (n = 3); values are mean ± SEM. Different letters indicate significant differences at P <0.05.

### 3.3 Crayfish growth and gonad development at different light intensities

Light intensity significantly affects crayfish growth (**Figure 4A**). Higher crayfish weights were obtained at 50% RLI compared to controls (100% RLI group) (P <0.05), but not at 20%; survival rate followed a similar trend. At 50% RLI, nearly 50% of crayfish gonads were at stage I–III, about twice as many as in controls, while for the 20% RLI treatment, gonad development was similar in composition to that of controls (**Figure 4B**).

**Figure 4:**
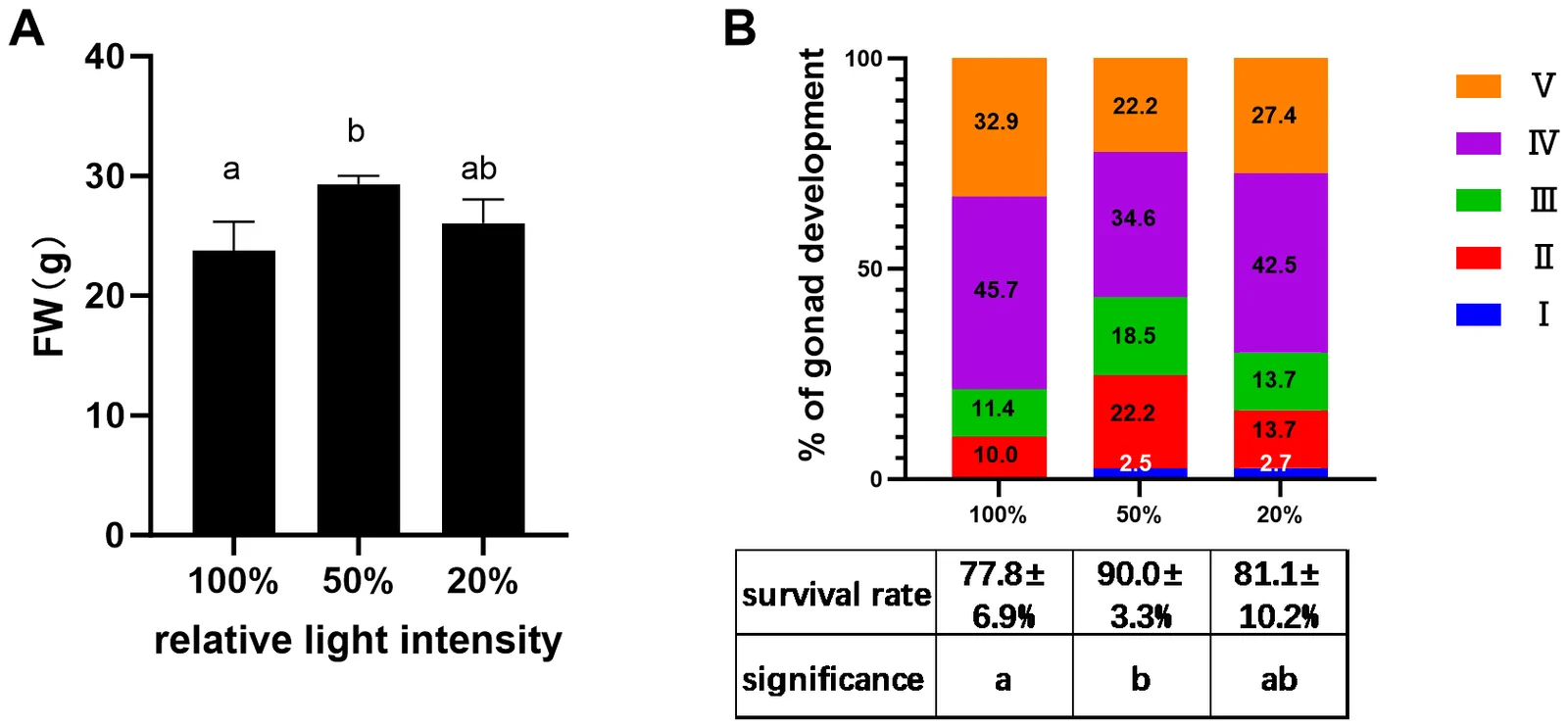
Effect of light intensity on growth and gonad development in crayfish, *Procambarus clarkia*. **(A)** The effect of light intensity on crayfish growth (FW, final weight); all values from three replicate tanks per treatment (n = 3) (mean ± SEM); **(B)** distribution of gonad developmental stages and survival rate at different light intensities (I, non-development; II, early developmental; III, previtellogenic; IV, vitellogenic; V, mature ovaries). Groups labeled with different lowercase letters indicate statistically significant differences at the 0.05 level.

### 3.4 Crayfish VTG metabolism at different light intensities

Hemolymph VTG concentrations were significantly higher in the control group (100% light intensity) than in the other two light-intensity groups (P <0.05) (**Figure 5A**). Hepatopancreatic vtg mRNA expression was significantly higher than that in the ovary in all treatments (**Figures 5B, C**). In gonad tissue, levels of vtg mRNA exhibited a statistically significant decline in response to a reduction in light intensity (P <0.05), but there was no significant difference in expression between treatments in hepatopancreatic tissues (**Figures 5B, C**). With decreased light intensity, VIH mRNA expression decreased significantly (P <0.05), but there were no significant differences in MIH between treatments (**Figures 5D, E**).

**Figure 5:**
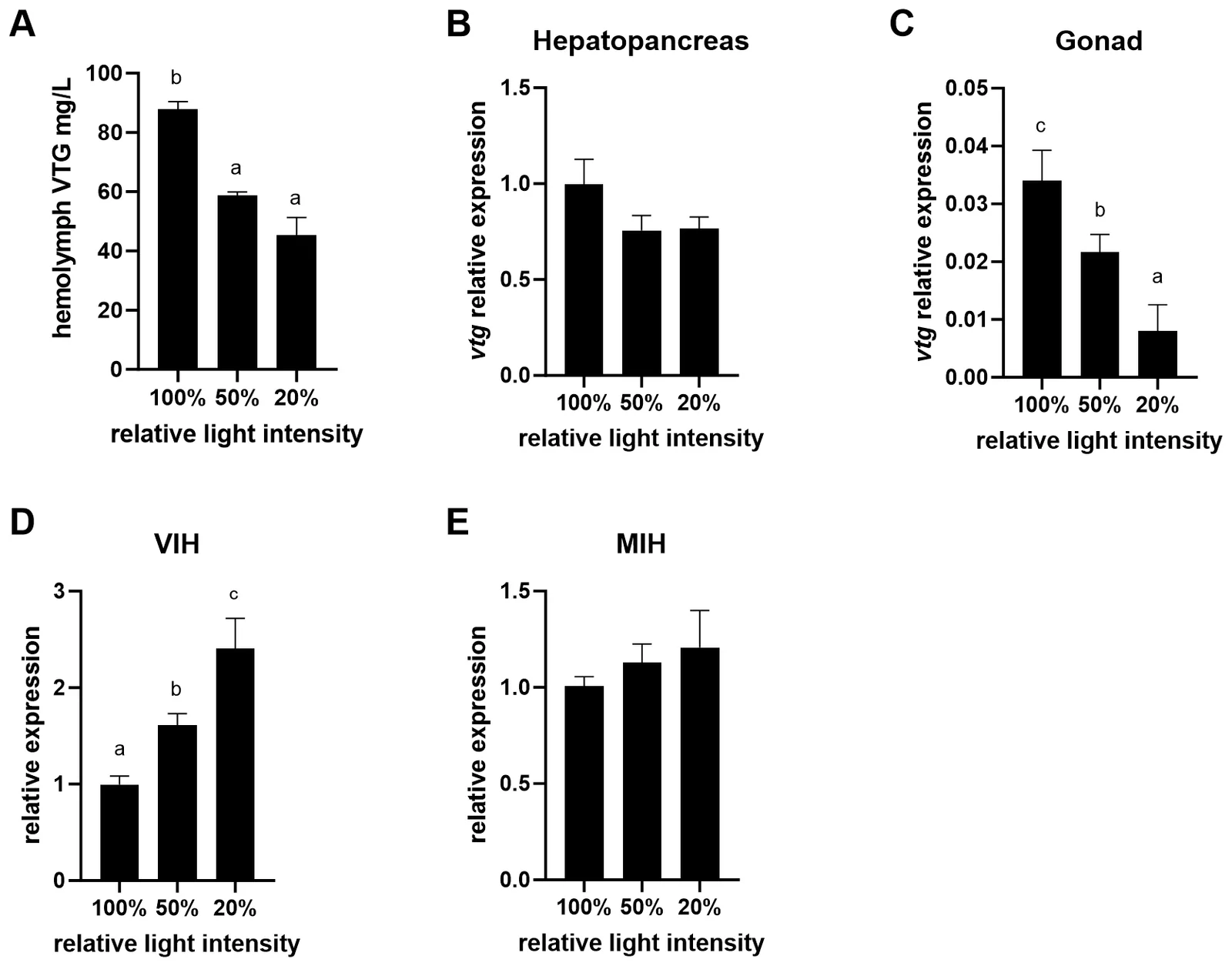
Effect of light intensity on VTG metabolism in crayfish, Procambarus clarkii: **(A)** Effect of light intensity in crayfish on hemolymph VTG content; **(B, C)** relative expression of *vtg* in the hepatopancreas **(B)** and gonad **(C)**; **(D, E)** relative expression of *vih* and *mih* in XO–SG compared to the amounts expressed at 100% relative light intensity. All values from three replicate tanks per treatment (n = 3) (mean ± SEM). Groups labeled with different lowercase letters indicate statistically significant differences at the 0.05 level.

## 4 Discussion

We sought to determine how temperature and light intensity influenced the growth and development of gonad tissue in red swamp crayfish. The impacts of photoperiod and temperature on ovarian development in *P. japonicus* are interdependent rather than independent, and rely on a combination of the two factors (Matsuda et al., 2002).

Generally, under natural conditions, most crayfish gonad development occurs in summer, when water temperatures are high (Gong et al., 2012). Temperature can promote gonad maturation (Carmona-Osaldea et al., 2004; Delgado and Camacho, 2007; Pervaiz et al., 2015), and once water temperature exceeds 28 °C, gonad development in >50% of crayfish is more advanced than the previtellogenic stage. This suggests that warmer water temperature may advance the ovarian development of this species. We found that raising water temperature caused a significant increase in crayfish body weight, and to advance gonad maturation. In juvenile catfish, short-term heat stress (34–36 °C) can significantly inhibit gonad development (Byerly et al., 2005). Aforementioned studies have reported gonad development of catfish to improve at temperature conditions suitable for its growth, but beyond a certain temperature, gonad maturation to be inhibited, along with other physiological and metabolic processes. This is similar to our results, in which we report survival rate to reduce significantly at temperatures >28 °C, despite gonad developmental stage increasing. Therefore, additional environmental variables are needed to balance growth, gonad development, and survival at high temperatures.

Positive stimulation of oocyte development can be achieved by manipulating photoperiod in gray mullet (*Mugil cephalus*) (6L:18D) and carp (*Carassius carassius* L.) (8L:16D) (Kuo et al., 1974; Seymour, 2010). However, for gazami crab (*Portunus trituberculatus*), increasing light duration promotes gonad maturation (Hamasaki et al., 2010). Therefore, cumulative light exposure affects reproductive development in aquatic animals to varying degrees. Higher light intensities (3,000 lx) increased gonad maturation in whitespotted rabbitfish, *Siganus sutor* (Shirinabadi et al., 2013). We report rates of crayfish weight gain and survival at approximately 50% RLI (400–500 lx) to increase significantly compared with controls, the degree of gonad development to reduce, but differences in indexes under reduced light intensities to be insignificant (**Figure 4**). The findings indicate that at elevated temperatures, reducing light intensity can result in reduced gonad development in red swamp crayfish, while concurrently increasing rates of weight gain and survival.

Serum levels of VTG reflect gonad developmental stages in red swamp crayfish, which are characterized by rapid accumulation of VTG in oocytes during later stages (IV and V) (Kang et al., 2014). It has been demonstrated that VTG levels in serum increase in a temperature-dependent manner, with a significant elevation above 32 °C. We found that the proportion of gonadal development at different temperatures (**Figure 2B**) were positively correlated with the VTG content in hemolymph and the expression of VTG in hepatopancreas and gonads (**Figures 3A–C**), which implies that high temperature affects the elevation of the degree of gonadal maturation in relation to the synthesis of VTG in crayfish. Nevertheless, a notable decline in serum VTG at reduced light intensity suggests that decreased serum VTG contents at high temperatures may impede gonad development. In crustaceans, VTG synthesized by the hepatopancreas is transferred to the ovary in serum during gonad development, and at the same time, the ovary can also synthesize VTG (Cai et al., 2016). We demonstrate that for different temperature and light conditions, expression of VTG in the ovary is lower than in the hepatopancreas, indicating that VTG contents in serum are mainly influenced by synthesis in the hepatopancreas. Therefore, lowering light intensity at high temperature may slow gonad maturation by reducing VTG accumulation in the ovary.

Eyestalk excision has been used to promote gonad development in crustaceans. The XO–SG complex in the eyestalk is the main site of expression of genes in the CHH family that regulate growth, molt, and gonad development (Chen et al., 2014). Two members of this family, VIH and MIH, are key regulators of vitellogenesis and molting, with VIH mainly inhibiting vitellogenesis and MIH mainly inhibiting molting (Jayasankar et al., 2020). We report VIH to be significantly downregulated, and MIH to be significantly upregulated at water temperatures exceeding 28 °C, consistent with changes in gonad stage; however, these associations do not by themselves demonstrate a causal relationship. Consistent with these results, expression of VIH/GIH genes in Japanese swamp shrimp increased and then decreased with increased temperature, with expression significantly lower at 27 °C than at 17 °C (Chen et al., 2021). Although the effect of light on expression of genes in the CHH family in crustaceans is not well studied, MIH expression under moderate light levels in juvenile mud crabs is decreased significantly compared with expression in dark and high-light conditions (Qiao et al., 2015). At high water temperatures, with decreased light intensity, we report VIH gene expression to gradually increase, and MIH gene expression to show no significant effect. We speculate that at high temperatures, red swamp crayfish VIH is more sensitive to a decrease in light intensity, which inhibits ovarian VTG synthesis.

## 5 Conclusion

In summary, water temperature above 28 °C reduced crayfish growth and survival and increased the proportion of late-developmental ovarian stages. At 36 °C, decreasing relative light intensity to approximately 50% improved weight gain and survival and delayed gonad development; these effects were accompanied by reduced ovarian vtg expression and lower VTG accumulation in the ovary, consistent with the observed changes in VIH and MIH expression in the XO–SG complex. Because spawning, fecundity, and gamete quality were not measured, these conclusions are restricted to growth and ovarian developmental endpoints.

## Data Availability

The original contributions presented in the study are included in the article/supplementary material, further inquiries can be directed to the corresponding author/s.
